# Mouse model of carbon tetrachloride induced liver fibrosis: Histopathological changes and expression of CD133 and epidermal growth factor

**DOI:** 10.1186/1471-230X-10-79

**Published:** 2010-07-09

**Authors:** Tsutomu Fujii, Bryan C Fuchs, Suguru Yamada, Gregory Y Lauwers, Yakup Kulu, Jonathan M Goodwin, Michael Lanuti, Kenneth K Tanabe

**Affiliations:** 1 Division of Surgical Oncology, Massachusetts General Hospital Cancer Center and Harvard Medical School, Boston, USA; 2 Department of Pathology, Massachusetts General Hospital Cancer Center and Harvard Medical School, Boston, USA; 3 Division of Thoracic Surgery, Massachusetts General Hospital Cancer Center and Harvard Medical School, Boston, USA

## Abstract

**Background:**

In the setting of chronic liver injury in humans, epidermal growth factor (EGF) and EGF receptor (EGFR) are up-regulated and have been proposed to have vital roles in both liver regeneration and development of hepatocellular carcinoma (HCC). Chronic liver injury also leads to hepatic stellate cell (HSC) differentiation and a novel subpopulation of HSCs which express CD133 and exhibit properties of progenitor cells has been described in rats. The carbon tetrachloride (CCl_4_)-induced mouse model has been historically relied upon to study liver injury and regeneration. We exposed mice to CCl_4 _to assess whether EGF and CD133+ HSCs are up-regulated in chronically injured liver.

**Methods:**

CCl_4 _in olive oil was administered to strain A/J mice three times per week by oral gavage.

**Results:**

Multiple well-differentiated HCCs were found in all livers after 15 weeks of CCl_4 _treatment. Notably, HCCs developed within the setting of fibrosis and not cirrhosis. CD133 was dramatically up-regulated after CCl_4 _treatment, and increased expression of desmin and glial fibrillary acidic protein, representative markers of HSCs, was also observed. EGF expression significantly decreased, contrary to observations in humans, whereas the expression of amphiregulin, another EGFR ligand, was significantly increased.

**Conclusions:**

Species-specific differences exist with respect to the histopathological and molecular pathogenesis of chronic liver disease. CCl_4_-induced chronic liver injury in A/J mice has important differences compared to human cirrhosis leading to HCC.

## Background

Hepatocellular carcinoma (HCC) is the fifth most common cancer worldwide and is the third leading cause of cancer mortality [1]. Eighty percent of HCCs develop in the context of chronic liver diseases, as chronic liver injury generally induces liver fibrosis, followed by cirrhosis [2]. In an attempt to model this process, carbon tetrachloride (CCl_4_) has been widely used to experimentally induce liver injury in rodents. A single dose of CCl_4 _leads to centrizonal necrosis and steatosis [3], while prolonged administration leads to liver fibrosis, cirrhosis, and HCC [4]. CCl_4 _impairs hepatocytes directly by altering the permeability of the plasma, lysosomal, and mitochondrial membranes. Highly reactive free radical metabolites are also formed by the mixed function oxidase system in hepatocytes via CYP2E1, causing severe centrilobular necrosis [5,6]. This model has been used extensively to examine the pathogenesis of cirrhosis.

Liver fibrosis is the pathologic result of ongoing chronic inflammatory liver diseases and is characterized by hepatic stellate cell (HSC) proliferation and differentiation to myofibroblast-like cells, which deposit extracellular matrix (ECM) and collagen. Quiescent HSCs are vitamin A storing cells in the space of Disse, and they account for about 15% of the total number of liver cells [7]. The activation of HSCs is mediated by reactive oxygen species and various cytokines, including transforming growth factor (TGF)-β, tumor necrosis factor (TNF)-α, and platelet-derived growth factor (PDGF), as well as other factors which are released from the damaged hepatocytes and activated Kupffer cells [8]. The activated HSCs produce large amounts of ECM components, such as laminin and collagen type IV, in an accelerated fashion, resulting in fibrotic change of the liver. The number of HSCs was found to be increased in alcoholic liver disease and in other animal models of chronic liver disease [8]. HSCs are further characterized by their stellate-shaped morphology and expression of desmin and glial fibrillary acidic protein (GFAP). A novel subpopulation of HSCs in rats has been described to exhibit properties of progenitor cells and express CD133, originally thought to be a marker of endothelial progenitor cells (EPCs), hematopoietic stem cells, and other stem cells [9]. This finding gave rise to the hypothesis that CD133+ HSCs are up-regulated in chronically injured liver.

Epidermal growth factor (EGF), a polypeptide mitogen, and its tyrosine kinase receptor (EGFR) have been proposed to have vital roles in liver regeneration and transformation [10,11]. EGF and EGFR are highly elevated in human cirrhotic livers [12]. However, to the best of our knowledge, the expression of EGF and EGFR in the injured liver in mouse models has not been fully investigated.

In the current study, we exposed mice to CCl_4 _to create fibrosis, cirrhosis, and HCC and assessed histopathology, EGF expression, and HSC populations. We observed that CD133+ HSCs are recruited during chronic liver injury in CCl_4_-treated mice. Multiple HCCs were found in the livers of all mice after 15 weeks of CCl_4 _treatment; however, the pathological findings and EGF expression patterns in the injured liver were different from those previously reported in humans, suggesting that species-specific differences exist with respect to the histopathological and molecular pathogenesis of chronic liver injury.

## Methods

### Animals and Experimental Design

**Figure 1:**
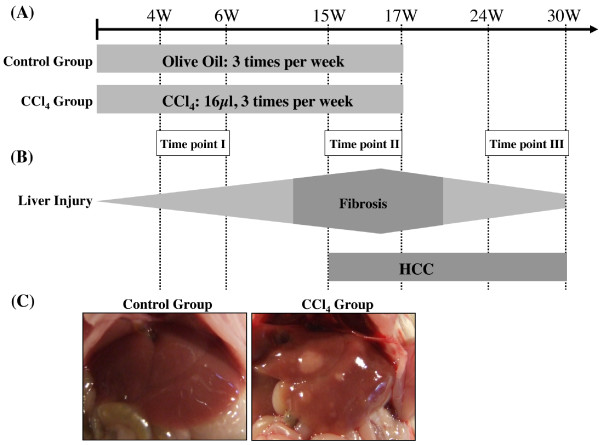
**Flow diagram depicting treatment of strain A/J mice with carbon tetrachloride (CCl_4_)**. (A) Schema. Mice were treated three times per week for 17 weeks by oral gavage with 0.04 cc of a 40% solution of CCl_4 _in olive oil or the vehicle control. (B) Experimental observations. Fibrosis was evident 15 weeks after initial CCl_4 _treatment, and hepatocellular carcinomas (HCCs) were identified macroscopically in 100% of mice after 17 weeks. Mice were classified into three groups depending on the time of sacrifice (Time point I (4-6 weeks), Time point II (15-17 weeks), Time point III (24-30 weeks)). (C) Representative macroscopic pictures of livers from mice sacrificed at Time point III are shown.

Strain A/J male mice at approximately 5 weeks of age were purchased from Jackson Laboratory (Bar Harbor, ME). As shown in Figure 1, all mice were randomly assigned to two groups: a control group (n = 15) and a CCl_4 _group (n = 25). Mice were treated three times a week for 17 weeks with either 0.04 cc of a 40 percent solution of CCl_4 _(Sigma, St. Louis, MO) in olive oil or with vehicle by oral gavage. Mice were subsequently sacrificed at the indicated times after a one-week washout to eliminate acute effects of CCl_4_. The liver was sectioned and fixed in phosphate-buffered 10% formaldehyde for histological analysis. The remaining portions of the liver were collected in RNase-free tubes and snap-frozen in liquid nitrogen. Mice were classified into three groups, depending on the time of sacrifice: Time point I (4-6 weeks), Time point II (15-17 weeks), Time point III (24-30 weeks).

### Histopathological Examination

Each formaldehyde-fixed sample was embedded in paraffin, cut into 5 μm-thick sections and stained with hematoxylin-eosin (H-E), Masson's trichrome and Sirius red according to standard procedures. Those from CCl_4 _and control groups at Time point II were stained with antibodies for CD133, desmin and alpha-smooth muscle actin (α-SMA) (all from Abcam; Cambridge, MA) according to the manufacturer's instructions. All slides were reviewed by the same pathologist.

### Quantitative Real-Time Polymerase Chain Reaction

Quantitative real-time polymerase chain reaction (PCR) was carried out as described previously [11]. Briefly, total RNA was extracted from a small piece of frozen mouse liver using TRIzol (Invitrogen, Carlsbad, CA) according to the manufacturer's instructions.

The mRNA expression of EGF, EGFR, transforming growth factor-alpha (TGF-α), heparin-binding EGF-like growth factor (HB-EGF), amphiregulin (AREG), desmin, GFAP, CD133 and vascular endothelial growth factor receptor 2 (VEGFR2) was analyzed. The mRNA expression was normalized to the expression of GAPDH. Primer sequences were as follows: SEQ NO.1 EGF forward (NM_010113.1 87-106 bp) GGCTTGGAACTTTCCATCAA, SEQ NO.2 EGF reverse (NM_010113.1 333-314 bp) CAGGTCCTTCTGCACCTCTC, SEQ NO.3 EGFR forward (NM_207655.1 349-368 bp) GGCGTTGGAGGAAAAGAAAG, SEQ NO.4 EGFR reverse (NM_207655.1 471-452 bp) TTCCCAAGGACCACTTCACA, SEQ N0.5 TGF-α forward (NM_031199.1 2395-2414 bp) CAGGGAGCAACACAAATGGA, SEQ N0.6 TGF-α reverse (NM_031199.1 2491-2471 bp) AGCCTCCAGCAGACCAGAAA, SEQ NO.7 HB-EGF forward (NM_010415.2 1147-1166 bp) GAAAGCAGGATCGAGTGAGC, SEQ NO.8 HB-EGF reverse (NM_010415.2 1369-1350 bp) CTTGCGGCTACTTGAACACA, SEQ NO.9 AREG forward (NM_009704.3 739-758 bp) GACTCACAGCGAGGATGACA, SEQ NO.10 AREG reverse (NM_009704.3 987-968 bp) GGCTTGGCAATGATTCAACT, SEQ NO.11 desmin forward (NM_010043.1 1723-1742 bp) AGCTCAAGTCATCGCCCTTC, SEQ. NO. 12 desmin reverse (NM_010043.1 1801-1781 bp) GCAGATCCCAACACCCTCTC, SEQ NO.13 GFAP forward (NM_010277.1 1078-1097 bp)AACCGCATCACCATTCCTGT, SEQ NO.14 GFAP reverse (NM_010277.1 1214-1196 bp) ACCTCACCATCCCGCATCT, SEQ NO.15 CD133 forward (NM_008935.1 1698-1717 bp) GAAAAGTTGCTCTGCGAACC, SEQ NO.16 CD133 reverse (NM_008935.1 1893-1874 bp) TCTCAAGCTGAAAAGCAGCA, SEQ NO.17 VEGFR2 forward (NM_010612.1 3574-3593 bp) ATGCGGGCTCCTGACTACAC, SEQ NO.18 VEGFR2 reverse (NM_010612.1 3683-3664 bp) CCCAAATGCTCCACCAACTC, SEQ NO.19 GAPDH forward (NM_008084 543-562 bp) AACTTTGGCATTGTGGAAGG, SEQ NO.20GAPDH reverse (NM_008084 765-746 bp) ACACATTGGGGGTAGGAACA. All reactions were performed in duplicate, and the experiment was repeated to ensure reproducible results.

### Enzyme-Linked Immunosorbent Assay (ELISA)

Liver tissue samples were homogenized in RIPA Buffer (Boston BioProducts, Worcester, MA) containing protease inhibitors (Sigma). Homogenates were centrifuged twice at 10,000 × *g *for 30 minutes at 4°C, and the pellets were discarded. Protein concentration was determined using the bicinchoninic acid (BCA) method (Pierce Chemical Co., Rockford, IL). The protein level of EGF was examined using a Quantikine Immunoassay kit (R&D Systems Inc., Minneapolis, MN) according to the manufacturer's instructions. All samples were measured in triplicate.

### Western Blotting

Western blotting was carried out as described previously [11]. Briefly, cellular lysates (35 μg) were prepared in Laemmli's reducing sample buffer (Boston BioProducts), separated by electrophoresis on 4-20% polyacrylamide gradient gels (Cambrex Bio Science, Walkersville, MD). Membranes were incubated overnight at 4°C with primary antibodies raised against EGFR, phospho-EGFR (Tyr 1068) and proliferating cell nuclear antigen (PCNA) (all from Cell Signaling Technology, Beverly, MA) and then incubated with appropriate secondary antibodies for 1 h at room temperature. An antibody directed against β-actin (Abcam) was used to verify equal loading. Each western blot was repeated to ensure reproducible results.

### Statistical Analysis

The relative mRNA expression levels were calculated from the quantified data. An unpaired *t *test was used to analyze the differences, and a non-parametric test was used (Mann-Whitney U test) if the distribution was abnormal. All the statistical analyses were performed using Stat View 5.0 (Abacus Concepts, Berkeley, CA). Data are expressed as mean ± SD. *P *< 0.05 denoted the presence of a statistically significant difference.

## Results

### Histological Analysis

As shown in Figure 1A, mice either received CCl_4 _solution via oral gavage three times per week or olive oil alone as control. In the CCl_4 _group, mice became lethargic and lean, and their fur became lusterless. One or multiple nodules, as many as 10 occurring in one liver, were found macroscopically on the surface of the entire liver in 100% of mice after 15 weeks of CCl_4 _treatment (Figure 1B, C). Tumors size varied from 0.1 to 1.2 cm in diameter.

**Figure 2:**
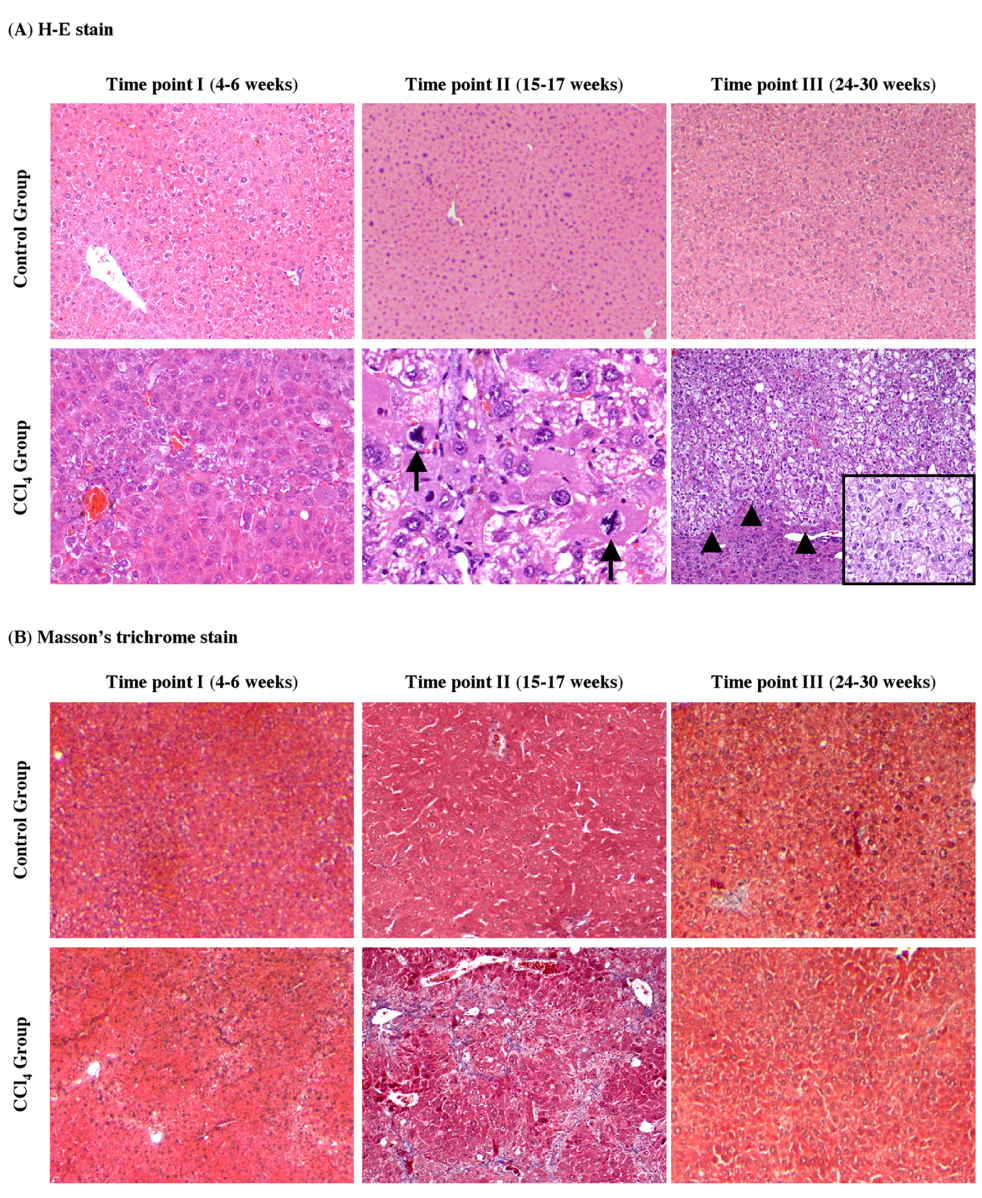
**Histopathological examination of mouse livers revealed fibrosis and HCC but no evidence of cirrhosis**. (A) Liver tissue from all control mice at each time point gavaged with olive oil alone revealed normal cellular architecture (upper, H-E, original magnification ×40). Liver tissue from CCl_4 _Group at Time point I revealed some damage of liver cells and centrilobular congestion with no infiltration of inflammatory cells (bottom left, H-E, original magnification ×200). Liver tissue from CCl_4 _Group at Time point II had increased mitotic activity (arrows), intense neutrophilic infiltration, extensive fatty changes and severe centrilobular necrosis (bottom middle, H-E, original magnification ×600). Liver tissue from CCl_4 _Group at Time point III revealed well-differentiated HCCs (arrowheads) with severe fatty changes (bottom right, H-E, original magnification ×40). High-power view (×200) of tumor cells is shown. (B) Masson's trichrome staining of the liver from all control mice at each time point revealed normal lobular architecture and a normal distribution of collagen (upper). Masson's trichrome staining of liver tissue from CCl_4 _Group at Time point II revealed extensive collagen deposition and pseudolobular formation, suggesting liver fibrosis (bottom middle). The degree of collagen deposition decreased at Time point III (bottom right). (original magnification ×40).

Liver samples were stained with H-E and Masson's trichrome to evaluate histopathologic changes and tissue fibrosis (Figure 2A, B). H-E staining of liver tissue from all control mice at each time point revealed normal cellular architecture (Figure 2A). Liver tissue from CCl_4 _Group at Time point I (4-6 weeks) demonstrated some cellular damage and centrilobular congestion with no infiltration of inflammatory cells. Liver tissue from CCl_4 _Group at Time point II (15-17 weeks) demonstrated increased mitotic activity, intense neutrophilic infiltration, extensive fatty changes and severe centrilobular necrosis. Well-differentiated HCCs and severe fatty changes were observed in livers from the CCl_4 _Group at Time point III (24-30 weeks). Masson's trichrome staining of the liver was performed to assess collagen distribution (Figure 2B). All control mice at each time point revealed a normal distribution of collagen. Extensive collagen deposition and pseudolobular formation was only evident in liver tissue from CCl_4 _Group at Time point II. When examined by a pathologist, the livers were determined to have bridging fibrosis (Ishak score 3-4). As previously mentioned, well-differentiated HCCs were confirmed microscopically in all mice after 15 weeks (Time point II) of CCl_4 _treatment; however, most notably, no evidence of cirrhosis (i.e. the presence of regenerative nodules) was identified in these livers, even in those with HCC. Given that the goal of this study was to create HCC in the setting of cirrhosis and HCCs had already developed in the absence of cirrhosis, CCl_4 _treatment was stopped at this point. Interestingly, the degree of inflammation and collagen deposition decreased at Time point III, suggesting that the liver repaired itself.

**Figure 3:**
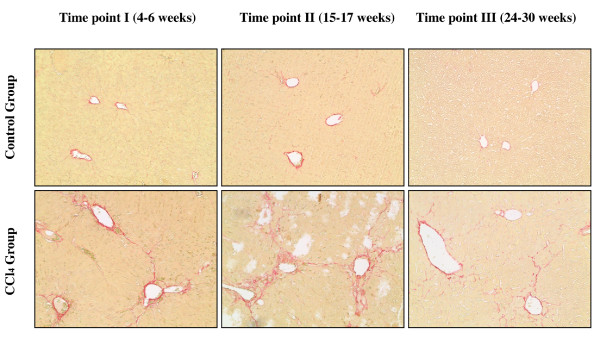
**Histopathological examination of mouse livers by Sirius red staining**. Sirius red staining of the liver from all control mice at each time point revealed normal lobular architecture and a normal distribution of collagen (upper). Sirius red staining of liver tissue from CCl_4 _Group at Time point II revealed extensive collagen deposition and pseudolobular formation, indicative of bridging fibrosis (bottom middle). The degree of collagen deposition decreased at Time point III (bottom right). (original magnification ×40).

To further assess the extent of fibrosis/cirrhosis after CCl_4 _treatment, Sirius red staining was performed on liver sections from each time point (Figure 3). The results were similar to the Masson's trichrome stains. Control mice had a normal distribution of collagen, whereas those treated with CCl_4 _at Time point I demonstrated early signs of fibrosis. Mice treated with CCl_4 _at Time point II demonstrated bridging fibrosis and those at Time point III had less collagen deposition suggesting again that the liver repaired itself.

### EGF and EGFR Expression

**Figure 4:**
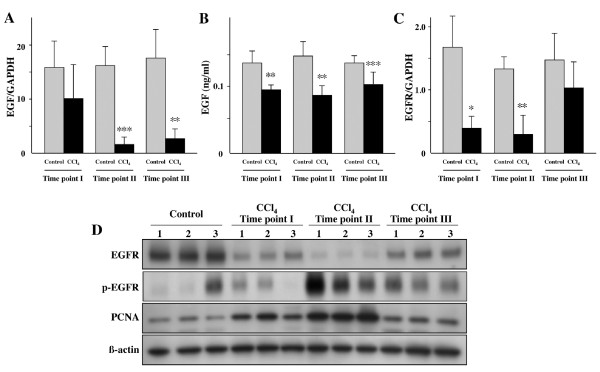
**EGF and EGFR expression was significantly decreased in mice treated with CCl_4_**. EGF expression was determined by (A) quantitative real-time PCR and (B) ELISA. The mRNA expression of (C) EGFR was examined by quantitative real-time PCR. (D) Western blot analysis of three representative liver tissues at each Time point for EGFR, p-EGFR (Y1068), PCNA and β-actin. Each value represents the mean ± SD. *P < 0.05, **P < 0.01 and ***P < 0.001 compared with respective control mice (Time point I: Control group n = 4, CCl_4 _Group n = 4; Time point II: Control group n = 2, CCl_4 _Group n = 5, Time point III: Control group n = 5, CCl_4 _Group n = 9).

We examined 11 control livers (Time point I, II, III; n = 4, n = 2, n = 5, respectively), 4 damaged but non-fibrotic livers (Time point I), 5 fibrotic livers (Time point II), and 8 non-cancerous livers (Time point III) for EGF mRNA expression by quantitative real-time PCR. EGF mRNA expression markedly decreased during liver injury (Figure 4A). To investigate EGF protein levels, ELISA for EGF was conducted in the same liver samples. EGF protein expression in the injured liver was significantly lower than in the normal liver (Figure 4B). EGFR mRNA expression also decreased significantly with increasing degree of liver injury (Figure 4C). Interestingly, we observed that total EGFR levels decreased with CCl_4 _administration while p-EGFR levels increased. The decrease in total EGFR in the presence of increased p-EGFR is likely a result of ligand-mediated receptor endocytosis [13]. This is consistent with decreased total EGFR protein expression observed in proliferating hepatocytes [14] which we confirmed by increased PCNA expression in response to CCl_4 _administration (Figure 4D). A correlation appears to exist then between p-EGFR levels and PCNA and therefore probably hepatocyte proliferation.

**Figure 5:**
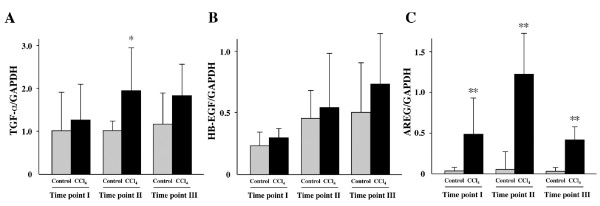
**TGF-α and AREG expression was significantly increased in mice treated with CCl_4_**. (A) TGF-α, (B) HB-EGF and (C) AREG expression were determined by quantitative real-time PCR. Each value represents the mean ± SD. *P < 0.05 and **P < 0.001 compared with respective control mice (Time point I: Control group n = 4, CCl_4 _Group n = 4; Time point II: Control group n = 2, CCl_4 _Group n = 5, Time point III: Control group n = 5, CCl_4 _Group n = 9).

Since we observed no increase in EGF expression, we examined the expression of several other EGFR ligands including HB-EGF, AREG and TGF-α, which has been reported to play an essential role in the regenerating liver [15]. TGF-α mRNA expression in livers treated with CCl_4 _was significantly higher than in control livers (Figure 5A), while HB-EGF levels did not change (Figure 5B). AREG was dramatically increased in livers treated with CCl_4 _(Figure 5C).

### CD133 Expression

**Figure 6:**
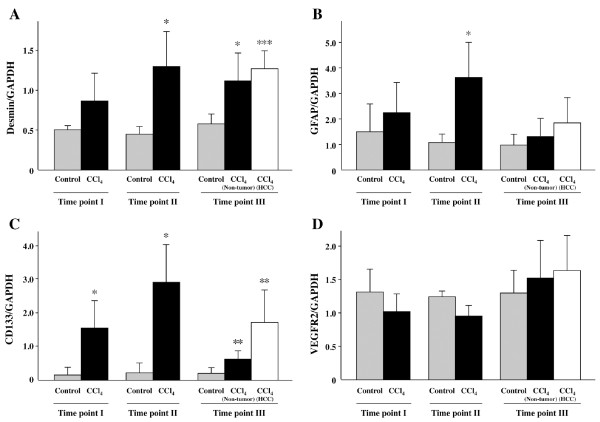
**CCl_4_-induced fibrosis in mice is associated with increased presence of hepatic stellate cells (HSCs)**. The presence of HSCs was determined by quantitative real-time PCR for the following markers: (A) desmin, (B) GFAP, and (C) CD133. The mRNA expression of these markers was significantly up-regulated in the fibrotic livers of CCl_4_-treated mice. Upon withdrawal of CCl_4_, the expression of CD133 returned to control levels in the surrounding normal tissues but remained elevated in the tumors (C). No increase was seen by quantitative real-time PCR for (D) VEGFR2, one of the surface markers of EPCs. Each value represents the mean ± SD. *P < 0.05, **P < 0.01 and ***P = 0.0001 compared with respective control mice (Time point I: Control group n = 4, CCl_4 _Group n = 4; Time point II: Control group n = 2, CCl_4 _Group n = 5, Time point III: Control group n = 5, CCl_4 _Group (Non-tumor part) n = 9, CCl_4 _Group (HCC part) n = 5).

**Figure 7:**
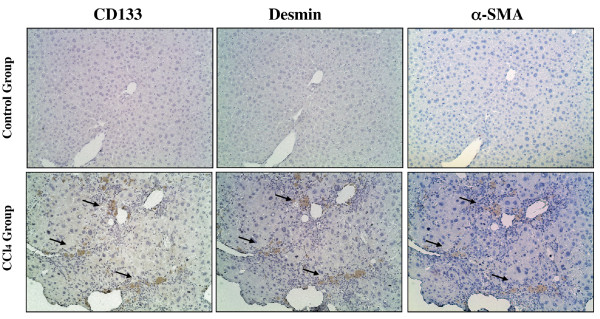
**CD133, desmin and α-SMA localize together in CCl_4_-treated mouse livers**. Liver sections from CCl_4 _and control groups at Time point II were stained with antibodies for CD133, desmin and α-SMA.

To evaluate HSCs in injured livers, quantitative real-time PCR was performed for desmin and GFAP, known to be representative markers of HSCs [16]. Increased expression of both desmin and GFAP mRNA was observed in the more extensively injured liver samples (Figure 6A, B). Furthermore, CD133 was also dramatically up-regulated in the livers of CCl_4_-treated mice (Figure 6C). Upon withdrawal of CCl_4_, the expression of CD133 returned to control levels in the surrounding normal tissues but remained elevated in the tumors. CD133 positive cells probably correspond to HSCs as an immunohistochemical study revealed that CD133, desmin and α-SMA (a marker for activated HSCs) localized to the same regions in CCl_4_-treated mouse livers (Figure 7). Further, the mRNA expression of VEGFR2, reported to be one of the surface markers of EPCs, was not altered (Figure 6D).

In order to exclude the possibility that CCl_4 _simply promotes CD133 expression in existing parenchyma cells, an *in vitro *study was also conducted. Hepa 1-6 (mouse hepatoma), THLE-5B (non-tumorigenic human liver), HepG2 (human hepatoblastoma) and SNU-182 (human hepatoma) cell lines were treated with 4 mM CCl_4 _for 24 hours, and CD133 mRNA expression was determined. CCl_4 _treatment did not increase CD133 expression in human or mouse cell lines or in non-tumorigenic or cancer cell lines (data not shown). Further, in this small sampling, no differences in CD133 expression were seen between non-tumorigenic and tumorigenic cell lines from the same species.

## Discussion

CCl_4_-induced toxicity and its mechanisms have been extensively investigated after oral administration to rodents. A large number of rat models have been reported to develop both liver cirrhosis and HCC using CCl_4 _or diethylnitrosamine. In the current study, we generated well-differentiated HCCs in the livers of all CCl_4_-treated strain A/J mice. Fibrosis but not cirrhosis was identified in these livers, even in those with HCCs. The cessation of oral gavage of CCl_4 _after 17 weeks may explain why cirrhosis did not develop although HCC clearly developed before any signs of cirrhosis. In previous reports of a rat model, liver cirrhosis induced by toxic agents preceded the development of HCC, like in humans [17,18]. In this study, HCCs were obviously generated in fibrotic, not cirrhotic, livers.

Many investigators have previously reported their findings in mice treated with CCl_4_, each with different mouse strains and CCl_4 _administration schedules. We therefore reviewed eight published reports of mouse models of liver "cirrhosis" induced only by CCl_4 _to assess fibrosis, regenerative nodules, and cirrhosis [19-26]. One report of a transgenic mouse model was also reviewed [27]. Of the nine models previously reported in the literature, only the model by Xue et al. shows incomplete cirrhosis (Ishak score = 5) with portal fibrosis, bridging fibrosis, and regenerative nodules [23,28]. Two other models show changes similar to ours [19,24], all the others showed only limited portal fibrosis and bridging fibrosis with no regenerative nodules. Thus, it may be more difficult to induce cirrhosis in mice.

The mechanism mobilizing the normally quiescent hepatocyte into mitogenesis in injured liver is poorly understood, although accumulating data suggest that EGF and TGF-α, specific ligands of EGFR, play a central role in initiating and/or sustaining the early growth response program [15,28,29]. We obtained evidence that both mRNA and protein expression of EGF significantly decreased during liver injury, but increased during repair after the withdrawal of CCl_4_. The transcription of EGF was shown to be extremely low in control livers but was highly elevated in cirrhotic livers of human patients, and EGF expression was increased significantly during the course of cirrhosis development in a rat model [12,30]. Therefore, histopathological changes and EGF expression patterns observed in this mouse model were different from those previously reported in rats and humans. Consistent with previously reported rat models of liver cirrhosis, EGFR expression was shown to decrease in this mouse study, whereas it has been reported to increase in human cirrhosis patients [31,32]. However, these results are difficult to interpret as decreased levels of total EGFR observed in animal models might be due to ligand-mediated receptor endocytosis in response to elevated signaling.

Combined with our histopathological analysis of previously published reports, our results indicate that there are species-specific differences in liver regeneration in response to cytokines and growth factors. Previous investigators have provided evidence of the differences in the response to hepatotoxicity in the livers of rodents. High levels of inhalation exposure to CCl_4 _resulted in liver cirrhosis in rats but in neither fibrosis nor cirrhosis in mice [33]. Furthermore, inflammation of the liver was almost absent in rats after common bile duct ligation but was more pronounced in mice [34]. It was also reported that the lack of circulating EGF in sialoadenectomized mice did not decrease the proportion of hepatocytes that replicate during liver regeneration after partial hepatectomy [35], whereas removal of the salivary glands in rats led to complete blockage of liver regeneration [36]. It would appear that other EGFR ligands might be critically involved in liver regeneration in mice instead of EGF [28,37]. For example, both TGF-α and AREG increased in injured livers in this study. TGF-α has previously been shown to increase in previous reports of rats and humans and AREG has previously been shown to participate in the development of mouse liver fibrosis [38]. Importantly, AREG has also been shown to contribute to the neoplastic phenotype of human HCC cells (Castillo 2006 Cancer Research) and therefore the CCL4 model could be used to further study the role of AREG in hepatocarcinogenesis.

We observed that the expression of both desmin and GFAP, known to be markers of HSCs, were significantly elevated in injured liver samples. This finding implicates increased numbers of HSCs in the livers of CCl_4_-treated mice. In a recent study, Kordes et al. demonstrated that 20-40% of HSCs expressed CD133 and exhibited properties of progenitor cells in rats [9]. Motivated by this report, we also examined CD133 expression in the injured livers of mice. The mRNA expression of CD133 was observed to be up-regulated in more injured liver as was desmin and GFAP. Further, immunohistochemical staining revealed that CD133, desmin and α-SMA localized together in CCl_4_-treated mice livers. CD133 has been recognized as a cell surface marker of stem/progenitor cells. However, the mRNA expression of VEGFR2, reported to be one of the markers of both early and mature EPCs was not associated with the degree of the liver injury. Our results indicated that CD133 positive cells probably correspond to HSCs, not EPCs, and that CD133+ HSCs increased through chronic liver injury in CCl_4_-treated mice. Finally, CD133 expression returned to control levels in the surrounding non-cancerous tissues but remained elevated in HCCs.

CD133+ cells have been identified in the ductular reactions of chronically damaged human livers [39]. CD133 has been implicated as a marker for cancer stem cells of epithelial origin [40], and consistent with this, CD133+ cells isolated from human HCC cell lines have been reported to have cancer stem cell properties [41,42]. Further, CD133 expression has been reported to be associated with poor disease-free survival in HCC patients but was negatively associated with HBsAg, implicating a non-viral origin of CD133 expression in HCC. Here, we have shown that a chemical toxicity induces CD133 expression in the liver. Overall, our results suggest an association between CD133 upregulation and tumor generation although details must be elucidated by further investigation.

Our results suggest that the use of CCl_4_-induced chronic liver injury in mice has important differences compared to human cirrhosis. In humans, HCCs typically develop within the setting of cirrhosis; however, in this study, no evidence of cirrhosis was seen, and HCCs developed within a setting of fibrosis. Furthermore, EGF expression decreased significantly during chronic liver injury, whereas it has been shown to increase during development of human cirrhosis. These results suggest that species-specific differences exist with respect to the histopathological and molecular pathogenesis of chronic liver disease. The dramatic up-regulation of CD133 is a notable finding, and the contribution of CD133+ HSCs to liver regeneration and tumor progression await further studies in rodents and humans.

## Abbreviations

EGF: epidermal growth factor; EGFR: epidermal growth factor receptor; HCC: hepatocellular carcinoma; HSC: hepatic stellate cell; CCl_4_: carbon tetrachloride; TGF: transforming growth factor; HB-EGF: heparin-binding EGF-like growth factor; AREG: amphiregulin; GFAP: glial fibrillary acidic protein; EPC: endothelial progenitor cell

## Competing interests

The authors declare that they have no competing interests.

## Authors' contributions

*Study Concept and Design: *TF, BCF and KKT. *Acquisition of Data: *TF, BCF, GYL, YK, JMG and SY. *Analysis and Interpretation of Data: *TF, BCF, GYL, YK, JMG, SY, ML and KKT. *Drafting of the Manuscript: *TF, BCF, GYL and KKT. *Statistical Analysis: *TF, BCF and KKT. *Obtained Funding: *BCF, YK and KKT. *Administrative, technical or material support: *TF, BCF, GYL, YK, JMG, SY, ML and KKT. *Study Supervision: *TF, BCF and KKT. All the authors have read and approved the final manuscript.

## Pre-publication history

The pre-publication history for this paper can be accessed here:

http://www.biomedcentral.com/1471-230X/10/79/prepub
